# Determinants of Performance in the Timed up-and-go and Six-Minute Walk Tests in Young and Old Healthy Adults

**DOI:** 10.3390/jcm9051561

**Published:** 2020-05-21

**Authors:** Gallin Montgomery, Jamie McPhee, Mati Pääsuke, Sarianna Sipilä, Andrea B Maier, Jean-Yves Hogrel, Hans Degens

**Affiliations:** 1 Musculoskeletal Science and Sports Medicine, Department of Sport and Exercise Sciences, Manchester Metropolitan University, Manchester M15 6BH, UK; J.S.McPhee@mmu.ac.uk; 2 Institute of Sport Sciences and Physiotherapy, Faculty of Medicine, University of Tartu, 51014 Tartu, Estonia; mati.paasuke@ut.ee; 3 Gerontology Research Center, Faculty of Sport and Health Sciences, University of Jyvsäkylä, FI-40014 Jyvsäkylä, Finland; sarianna.sipila@jyu.fi; 4 Department of Human Movement Sciences, Vrije Universiteit Amsterdam, Amsterdam Movement Sciences, 1081 BT Amsterdam, The Netherlands; andrea.maier@unimelb.edu.au; 5 Department of Medicine and Aged Care, The Royal Melbourne Hospital, The University of Melbourne, Victoria 3010, Australia; 6 Neuromuscular Investigation Center, Institute of Myology, Pitié-Salpêtrière Hospital, 73013 Paris, France; jy.hogrel@institut-myologie.org; 7 Musculoskeletal Science and Sports Medicine, School of Healthcare Science, Manchester Metropolitan University, Manchester M1 5GD, UK; H.Degens@mmu.ac.uk; 8 Institute of Sport Science and Innovations, Lithuanian Sports University, LT-44221 Kaunas, Lithuania; 9 University of Medicine and Pharmacy of Targu Mures, 540142 Targu Mures, Romania

**Keywords:** muscle, spirometry, ageing, physical functional performance

## Abstract

The aim of this study was to assess associations between performance in the timed up-and-go (TUG) and six-minute walk distance (6MWD) with physiological characteristics in young and old healthy adults. Thereto, we determined TUG, 6MWD, normalised jump power, centre of pressure displacement during 1-leg standing, forced expiratory volume in 1 s, percentage of age-predicted maximal heart rate (HR%) and height in 419 healthy young (men: 23.5 ± 2.8 years, women: 23.2 ± 2.9 years) and old (men: 74.6 ± 3.2 years, women: 74.1 ± 3.2 years) adults. Normalised jump power explained 8% and 19% of TUG in young (*p* = 0.025) and older men (*p* < 0.001), respectively. When fat mass percentage and age were added to normalised jump power, 30% of TUG was explained in older men (R^2^_adj_ = 0.30, *p* < 0.001 to 0.106). Appendicular lean muscle mass percentage (ALM%) and age were the best determinants of TUG for older women (R^2^_adj_ = 0.16, *p* < 0.001 to 0.01). HR% explained 17–39% of 6MWD across all groups (R^2^_adj_ = 0.17 to 39, *p* < 0.001). In conclusion, in men, jump power was a key determinant for TUG, while in old women only it was the ALM%. As HR% was the most important determinant of 6MWD, motivational bias needs to be considered in the interpretation of this test.

## 1. Introduction

The timed-up-and-go test (TUG) [1] was originally used to assess physical mobility in frail elderly individuals and was thought to represent a simple and effective means of evaluating balance, gait speed, and the ability to perform daily life tasks that are required for autonomy [1]. Since then, the TUG has been used as a reliable measure of physical function in a variety of populations [2,3,4,5,6] and has been recommended for identifying dynamic balance parameters [7,8,9] and the onset of physical disabilities [10]. Indeed, poor TUG performance has been related to higher recurrent fall prevalence [11,12,13], lower bone mineral density and higher fracture rates [14]. Perhaps an even stronger indicator is that balance mobility training improved TUG in older adults [15]. In light of this, the TUG has been suggested as a valid screening tool to identify balance deficits in older adults [16].

In addition to measures of balance, jump power normalised to body mass is strongly related to TUG and six-minute walk distance (6MWD), and more so than maximal force normalised to body mass [17,18,19]. In the oldest populations, performance in the TUG and 6MWD is also more closely related to lower limb extensor explosive force than maximal force [20] indicating that changes in the shortening velocity in addition to force loss do contribute to the lower TUG in old age [17]. This suggests that muscle contractile properties and force-generating capacity are both important for retaining physical function in old adults. As TUG is a widely used measure of physical function for a range of clinical populations, it is important to establish the determinants of TUG performance in healthy young and older adults.

The 6MWD has been used to predict maximal aerobic capacity in healthy middle-aged and older adults [21,22] and is reduced in geriatric patients with cardiopulmonary disease [23]. Other common uses include diagnosis of sarcopenia [24], a measure of maximal aerobic capacity in lung disease and spinal muscular atrophy patients [25,26,27], and an indicator of all-cause mortality risk in older adults [28]. In young healthy individuals, standing height and changes in heart rate accounted for 31% to 38% of the variance in the 6MWD. Also age, height, heart rate change and pre and post-test systolic and diastolic blood pressure showed significant associations with the 6MWD [29,30]. When factors such as body mass index, lower limb muscle power, habitual physical activity, and strength were added, a larger proportion of the variance in 6MWD could be explained in severely obese middle-aged adults [19,31,32,33]. Ventilatory function has also been found to positively correlate with the 6MWD, in healthy older and pulmonary disease patients [34,35,36]. In addition, in older adults, poor balance is associated with lower walking speeds [37]. The determinants of 6MWD have been reported in previous clinical populations but the determinants of 6MWD in healthy young and older adults are currently unclear.

Previous studies have been limited by small homogeneous populations and have assessed a limited number of variables to explain the TUG and 6MWD. Few studies have analysed whether the determinants of performance in the TUG and 6MWD differ between young and old men and women. Further investigation is therefore warranted in a larger multicentre cohort of healthy young and old men and women applying an integrative analysis of determinants of physical function across the lifespan to inform clinical practice. Specifically, the objective of this study was to examine associations between performance in the TUG and 6MWD with normalised jump power, balance, lung function, percentage of age-predicted maximal heart rate (HR%) and height in a large multicentre cohort of healthy young and old men and women. We hypothesised that normalised jump power and balance are determinants of performance in the TUG and that normalised jump power, lung function, and HR% [29] are determinants of performance in the 6MWD.

## 2. Experimental Section

### 2.1. Participants

Four hundred and nineteen participants were recruited from the European multi-centre MYOAGE cohort [38]. Participants were healthy young adults (18–30 years) and older (69–81 years) men and women. Testing was undertaken from 2009 to 2012 across four European institutions [Leiden, The Netherlands (35 young, 75 old participants); Paris, France (35 young; 70 old participants); Tartu, Estonia (38 young, 61 old participants); Jyväskylä, Finland (35 young, 70 old participants)]. Potential participants were excluded if they suffered from known musculoskeletal, metabolic, cardiovascular (except controlled hypertension), severe chronic obstructive pulmonary disease defined as GOLD stages 3 and 4, neurological or mental conditions, or used medication as indicated in previous work [38]. Participants with a body mass index <18 and >32 kg/m^2^ were also excluded along with those undertaking competitive sports (except recreational sports participation). Participants avoided strenuous exercise 48 h prior to the testing session and refrained from smoking two hours before the testing session. All studies were approved by the respective local ethical committees (Leiden University Medical Center, P10.060, May, 31 2010; CPP Ile-de-France VI, 2010-A00614-35, April, 8 2011; University of Tartu, 189M-12, January, 28 2010; Ethical Committee of Central Finland Health Care District, March, 2010) and adhered to the 1964 Helsinki declaration and its later amendments or comparable ethical standards. Informed consent was obtained from all individual participants included in the study and all participants were medically screened prior to participation.

### 2.2. Anthropometrics

Standing height (m) was measured to an accuracy of one millimeter whilst body mass was measured to an accuracy of 0.1 kg. Body mass index (BMI) was calculated as mass/height^2^ (kg/m^2^).

### 2.3. Dual-Energy X-ray Absorptiometry (DEXA)

Following a 12-h overnight fast, body composition was assessed using whole-body dual-energy X-ray absorptiometry (Finland—Lunar Prodigy, version en-Core 9.30; Estonia–Lunar Prodigy Advanced, version en-Core 10.51.006; France—Lunar Prodigy, version en-Core 12.30; GE Healthcare, Chalfont St Giles, UK). DEXA scans were performed by a trained technician according to the manufacturer’s quality control procedures including daily calibration. After manual offline adjustment, whole-body lean mass and fat mass were calculated. Lean mass was calculated as:

lean mass = total mass − fat mass − (1.82 × BMC)

Appendicular lean muscle mass (ALM) was also given as ALM/height^2^ and areas were demarcated as described previously [17].

### 2.4. Balance

The majority of participants undertook a balance assessment (*n* = 309), where postural sway was measured as centre of pressure displacement during one-leg standing with eyes open as measured from force platform data (Finland—Good Balance, Metitur, Finland; Estonia—Kistler, Winterthur, Switzerland; France—AMTI OR6-7, Watertown, MA, USA) [39]. The participant was asked to stand on one leg for a maximum of 30 s (contralateral leg maintained 5 cm from the ground) or until the test was stopped due to them moving their arms or touching the floor with their contralateral leg. Trial duration was determined as the time that the participant remained in the required stance. If the trial duration lasted 30 s, the trial was ended and classed as the participant’s best trial. Participants were barefoot and asked to stand quietly, hands by their side and visually focus on a black circle target (0.15 m in diameter) situated 3 m away aligned at eye level. Centre of pressure displacement (mm) was expressed as the root mean square of the centre of pressure displacements in the mediolateral direction (COP-ML_d_).

### 2.5. Six-Minute Walk Distance and Heart Rate

For the 6MWD, participants were instructed to complete as many 20-m laps (25-m in France) as possible in six minutes without running and the distance covered recorded [40]. Verbal encouragement was given every minute during the test. Heart rate was recorded during the test (Polar Electro, Oy, Finland) and the final heart rate upon completion of the six minutes was given as a percentage of the age-predicted maximum heart rate (HR%) using the following formula [41]:

HR% = [final heart rate/(220 − age in years)] × 100

### 2.6. Timed up-and-go

The TUG test involved getting up from a standard chair without armrests, walking around a cone 3 m in front of the chair and returning to the original sitting position as quickly as possible without running [1]. The test was initiated with a verbal “go” instruction from the investigator, and the time taken to complete the test was recorded. After a familiarisation attempt, three recorded efforts were undertaken with one minute rest intervals. Verbal encouragement was given in the rest intervals to promote faster tests. The quickest of the three attempts was recorded and used in the analysis.

### 2.7. Muscle Power

Leg extension power was assessed with a maximal effort countermovement vertical jump on a force platform (France: AMTI OR6-7, Watertown, MA, USA; Estonia: Kistler, Switzerland; Finland: custom built force platform). Three maximal-effort countermovement vertical jumps were performed with a 1-min rest between efforts. Vertical ground reaction force was recorded throughout the movement at 1000 Hz. Maximum power was calculated using the following equations from the vertical force trace (Fz) and body mass of the participant (m) with acceleration due to gravity (*g*) at a constant of 9.81 m/s^2^. Instantaneous vertical acceleration (a) was calculated and integrated to obtain instantaneous vertical velocity (v) and then power (P) was calculated from the instantaneous force and vertical velocity [42]:

Fz = m·a

$$v=\int_{0}^{t}a\left(t\right)dt=\int_{0}^{t}\left[\frac{Fz\left(t\right)}{m}-g\right]dt$$

P = Fz·v

The maximum power generated during the take-off phase of the three-countermovement vertical jumps was recorded, normalised to the body mass of the participant and used in the final analysis.

### 2.8. Spirometry

Participants completed three maximal spirometry tests whilst seated with their hips and knees flexed at 90° and wore a nose clip throughout the procedure. Participants were instructed to “blow into the mouthpiece as forcefully and as quickly as possible” and to continue blowing until no further air could be expelled (SpiroStar DX and Spiro2000 software, Medikro, Kuopio, Finland. Micro Medical Spiro USB spirometer and Spida 5 software, Cardinal Health, Dublin, OH, USA). Spirometric pulmonary function was not undertaken at the Estonia site. Forced expiratory volume in 1 s (FEV_1_) was recorded in litres according to the criteria of the American Thoracic and European Respiratory Society [43] and the highest recorded FEV_1_ value was used in the final analysis.

### 2.9. Statistical Analyses

Differences in participant characteristics by age-group and sex were formally tested using two-way between-measures analysis of variances (ANOVAs) (2 age groups × 2 sexes). All participant characteristics, body composition, and physiological variables were then normalised to the average value of the young male population in each individual country to adjust for any systematic differences between countries. Data were formally tested and visually inspected for independence, linearity, normality of residuals, multi-collinearity and homoscedasticity to ensure suitability for entry into multivariate regression analyses. Relationships between each body composition and physiological variable and performance in the two physical function tests (TUG and 6MWD) were first evaluated using Pearson’s product–moment correlation coefficient stratified by age-group and sex. Those variables that had a bivariate correlation with statistical significance of *p* < 0.1 were then selected for inclusion in multivariate regression models. Variables were entered into the models in order of the Pearson’s product–moment correlation coefficient (largest first). In age-group and sex-specific multivariate regression models, the most parsimonious model was identified as the model with the highest explained variance (R^2^_adj_), separately across young and old, men and women. Those body composition and physiological variables included in the most parsimonious model for any one of the separate subgroups (i.e., young-men, young-women, older-men, older women) were then included together in a final multivariate regression model that was run in each of the four sub-groups to highlight the different outcomes for age and sex groupings. The level of statistical significance was set at *p* < 0.05. All analyses were performed using R (R Foundation for Statistical Computing 2019, v3.6.1, Vienna, Austria).

## 3. Results

### 3.1. Participant Characteristics

Table 1 shows the characteristics of the different age and sex groups. There was a significant age-by-sex interaction for lean mass (*p* = 0.010), appendicular lean muscle mass (*p* = 0.001), appendicular lean muscle mass percentage (*p* = 0.009), ALM/height^2^ (*p* = 0.046), normalised jump power (*p* < 0.001), FEV_1_ (*p* < 0.001), and 6MWD (*p* = 0.032), which is reflected by a larger absolute age-related decline in men than women (Table 1). There was a significant age-by-sex interaction for fat mass percentage (*p* = 0.037), which is reflected by a larger age-related increase in men than women (Table 1).

In a separate analysis, values were converted to a percentage of the sex-matched average (data for women were expressed as a percentage of the average young woman, data for men were expressed as a percentage of the average young man in each country). This showed that there were no significant age-by-sex interactions, indicating a similar age-related percentage decline in men and women. There was, however, a significant age-by-sex interaction for fat mass percentage (*p* < 0.001), which was reflected by a larger age-related increase in fat mass percentage in men than in women.

### 3.2. Bivariate Regression Analyses

Bivariate associations between body composition variables, physiological variables and the timed up-and-go test and the six-minute walk distance are presented in Table 2.

### 3.3. Multivariate Regression Analyses

#### 3.3.1. Timed up-and-go

Normalised jump power as a single determinant explained 8% and 19% of the variance in TUG performance for young men (β = −0.24, *p* = 0.025) and older men (β = −0.88, *p* < 0.001), respectively, (Table 3). Whilst, normalised jump power as a single determinant explained 6% of the variance in TUG for older women (β = −0.46, *p* = 0.008), ALM% was a stronger single determinant and explained 13% of the variance in TUG for older women (β = −0.92, *p* < 0.001). Normalised jump power (β = −0.78, *p* < 0.001), fat mass (%) (β = 0.15, *p* = 0.001) and age (β = 0.01, *p* = 0.106) were the best determinants of TUG for older men (R^2^_adj_ = 0.30, *p* < 0.001 to 0.106), whereas ALM% (β = −0.82, *p* < 0.001) and age (β = 0.01, *p* = 0.01) were the best determinants of TUG for older women (R^2^_adj_ = 0.16, *p* < 0.001 to 0.01). There were no significant determinants of TUG performance for young women. 

When all explanatory independent variables were combined in a separate multivariate regression model stratified by age and sex, there were no significant determinants of TUG performance for young men, and only height was a significant determinant of TUG performance for young women (β = −1.00, *p* = 0.028) (Table 4). Normalised jump power (β = −0.75, *p <* 0.001) and fat mass (%) (β = 0.15, *p <* 0.001) were determinants of TUG performance in older men and fat mass (%) was a determinant of TUG performance in older women (β = 0.09, *p =* 0.014).

#### 3.3.2. Six-Minute Walk Distance

HR% was the strongest single determinant of 6MWD across all young and old, men and women (R^2^_adj_ = 0.17 to 39, *p* < 0.001), (Table 5). HR% (β = 0.31, *p* < 0.001) and lean mass (%) (β = 0.34, *p* = 0.01) were the best determinants of 6MWD in young men, whereas HR% (β = 0.35, *p* < 0.001) and COP-ML_d_ (β = 0.07, *p* = 0.010) were the best determinants of 6MWD in young women.

HR% (β = 0.29, *p* < 0.001), normalised jump power (β = 0.34, *p* < 0.001), lean mass (%) (β = 0.16, *p* = 0.169) and FEV_1_ (β = 0.11, *p* = 0.102), were the best determinants of 6MWD in older men (R^2^_adj_ = 0.53, *p* < 0.001 to 0.169). However, HR% (β = 0.32, *p* < 0.001), appendicular lean muscle mass percentage (β = 0.49, *p* < 0.001), FEV_1_ (β = 0.10, *p* = 0.076) and age (β = −0.01, *p* = 0.001) were the best determinants of 6MWD in older women (R^2^_adj_ = 0.60, *p* < 0.001 to 0.076). 

When all explanatory independent variables were combined in a separate multivariate regression model stratified by age and sex, only HR% was a significant determinant of 6MWD for young men (β = 0.33, *p* = 0.004), and only HR% (β = 0.55, *p* < 0.001) and lean mass (%) (β = 0.54, *p* = 0.008) were significant determinants of 6MWD for young women (Table 6). HR% (β = 0.42, *p <* 0.001), lean mass (%) (β = 0.27, *p =* 0.025) and age (β = −0.01, *p =* 0.001) were determinants of 6MWD in older men whereas HR% (β = 0.26, *p <* 0.001), normalized jump power (β = 0.23, *p =* 0.047) and age (β = −0.01, *p =* 0.027) were determinants of 6MWD in older women.

## 4. Discussion

The main observation of the present study was that normalised jump power was the main determinant of TUG in men, irrespective of age. In old, but not young women, the main determinant of TUG was the ALM%. The largest determinant of 6MWD was HR%, which suggests that motivational bias needs to be considered when interpreting this test. Indeed, it has been suggested that HR% serves as a measure of physical effort [29].

### 4.1. Timed up-and-go

Our results show that normalised jump power alone can determine 19% of the variance in TUG for older men, which is supported by previous studies that also show that lower limb power can explain 14% and 22% of TUG or a similar functional task for older people and severely obese adults, respectively [18,19]. Normalised jump power explained a comparable percentage of TUG for older men only, where in the other studies only older frail or obese people were studied. Indeed, in young men, normalised jump power determined 8% of the TUG, and in both young and older women, normalised jump power was not related to TUG. Our analysis showed that there were different physiological determinants of TUG dependent upon the age and sex of the participants.

Power is the product of force and velocity and is indeed to a large extent determined by maximal force production [44]. It is, therefore, no surprise that other measures of lower limb maximal muscle function have also found to explain TUG including explosive and maximal force production (7 to 8%) [8,19]. However, lower limb muscle power is more closely related to performance in physical function tests than maximal force production [45,46]. A large proportion of physical function is explained by jump take-off velocity (an integral component of power production) during a counter movement jump, with older participants showing lower physical function along with lower jump take-off velocity [17] which may be attributable to a reduced muscle shortening velocity in old age. A lower shortening velocity may be related to a selective type II fibre atrophy, slowing of the contractile properties of muscle fibres, and increased tendon compliance [47]. Thus, a lower muscle power and TUG score in old age are likely attributable to both muscle weakness and slowing of muscle contractile properties.

For young women, there were no significant determinants of TUG in the parsimonious model, which suggests that as young women are not affected by age-related declines in the measured variables, TUG performance is largely determined by other factors that were not measured in this study. For older women ALM% and age explained 16% of the variance in TUG. Body composition appears to be an important factor for TUG, where fat mass (%) explains 10% of the variance for older men and ALM% explains 13% of the variance in older women. A greater proportion of lean mass is likely to contribute to increasing propulsive forces, which may result in an improved TUG, whereas a greater proportion of fat mass will likely hinder TUG due to the larger mass component that reduces propulsive forces relative to body mass [17]. The combination of explanatory variables highlights these age and sex-related differences (Table 4), for which the only significant determinant variables are; height for young women, normalised jump power and fat mass (%) for older men, and fat mass (%) for older women. The significance of standing height for young women is probably due to taller individuals displaying greater stride lengths and consequently higher walking speeds [48,49,50,51]. Contrary to our initial hypothesis, COP-ML_d_ did not relate to TUG for any of the groups.

### 4.2. Six-Minute Walking Distance

The current results indicate that HR% explains 17 to 39% of the variance in the 6MWD across young and old, men and women. The HR% can serve as a measure of physical effort as a lower HR% is associated with a lower 6MWD, irrespective of sex and age. This indicates that the level of engagement with the test has a strong impact on the 6MWD, similar to previous observations [29].

Interestingly there was a correlation with relative measures of lean mass and 6MWD for young men, older men and older women [17,52], that has been shown previously for older men and women, and in older men respectively. We have also shown this association in young men, however, and this is most likely due to the association between walking speed and physical fitness.

Normalised jump power explained an additional 4% of the variance in the 6MWD for older men only. Interestingly, normalised jump power was not a significant determinant of 6MWD in any other group. Lower limb power has shown a strong correlation with 6MWD in mobility-limited older individuals whereas aerobic capacity has shown no relation, suggesting that lower limb power may become increasingly important in limiting the 6MWD in populations with mobility impairments [31]. The significance of power as a limiting factor is illustrated by the improvements in the 6MWD and gait speed after strength and power training [53,54], although in our study, normalised jump power has a small, but significant, association with 6MWD only in older men.

FEV_1_ explains an additional 6% of the variance in 6MWD for older women and 5% in older men although not significant. This is noteworthy, as irrespective of physical activity level FEV_1_ shows an age-related decline that may limit aerobic capacity with advancing ageing [43]. The age-related decline in FEV_1_ may be particularly important when considering that in older adults declines in resting lung function can occur with no changes in maximal heart rate over a six year period [55].

### 4.3. Limitations

The strength of this study was that healthy young and older volunteers were recruited and our work thus represents the determinants of performance during healthy ageing. In addition, this study had a cross-sectional design and can only highlight associations between physical function tests and physiological variables. To establish a causal effect of the measured variables on physical function, intervention studies are necessary. The multi-centre design may have meant that testing protocols were not exactly identical at different sites. To minimise this effect, (1) staff at all sites were trained to deliver the same protocols by the same experienced researcher and all equipment was calibrated according to the manufacturer’s requirement and (2) all data from a study centre were normalised to the average data from the young men in that centre.

## 5. Conclusions

In conclusion, in men, jump power was a key determinant for TUG, while in old women, it was only the ALM%. The largest single determinant of the 6MWD was HR%, which explained 17 to 39% of the variance in 6MWD across young and old men and women. When HR% was combined with normalised jump power (older men only), relative measures of body composition, FEV_1_ and age, 53 to 60% of the variance in 6MWD was explained in older men and older women respectively. As HR% was the most important determinant of 6MWD, motivational bias needs to be considered in the interpretation of this test. It is important to consider that individuals with low muscle power or individuals demonstrating a low level of effort are at risk of lower functional performance in a clinical setting.

## Figures and Tables

**Table 1:** Characteristics of study participants.

	*n*	Young Men	*n*	Young Women	*n*	Older Men	*n*	Older Women	Age	Sex	Age-Sex Interaction
Age (years)	66	23.5 ± 2.8	77	23.2 ± 2.9	138	74.6 ± 3.2	138	74.1 ± 3.2	<0.001	0.150	0.854
Body mass (kg)	66	76.1 ± 10.0	77	62.3 ± 9.1	138	77.9 ± 10.3	138	64.9 ± 9.6	0.008	<0.001	0.675
Height (m)	66	1.81 ± 0.06	77	1.67 ± 0.07	138	1.73 ± 0.06	138	1.61 ± 0.07	<0.001	<0.001	0.294
BMI (kg/m^2^)	66	23.3 ± 2.7	77	22.4 ± 2.8	138	25.9 ± 2.8	138	25.1 ± 3.8	<0.001	0.005	0.882
Fat mass (kg)	65	13.1 ± 5.6	76	18.5 ± 5.8	133	19.9 ± 6.5	136	23.1 ± 6.8	<0.001	<0.001	0.097
Lean mass (kg)	65	60.5 ± 7.5	76	41.7 ± 5.7	133	55.5 ± 6.3	136	40.1 ± 5.6	<0.001	<0.001	0.010 *
ALM (kg)	65	27.9 ± 3.4	76	18.4 ± 2.8	133	24.3 ± 3.1	136	16.8 ± 2.7	<0.001	<0.001	0.001 *
ALM/height^2^ (kg/m^2^)	65	8.5 ± 0.9	76	6.6 ± 0.8	133	8.1 ± 0.8	136	6.5 ± 0.7	0.005	<0.001	0.046 *
Fat mass (%)	65	16.8 ± 6.1	76	29.3 ± 5.9	133	25.2 ± 5.7	136	35.1 ± 6.5	<0.001	<0.001	0.037 *
Lean mass (%)	65	79.8 ± 6.3	76	67.6 ± 6.1	133	72.0 ± 6.4	136	62.3 ± 6.8	<0.001	<0.001	0.055
ALM%	65	36.8 ± 3.3	76	29.8 ± 2.7	133	31.4 ± 3.0	136	26.0 ± 3.2	<0.001	<0.001	0.009 *
Timed up-and-go (s)	65	4.9 ± 0.9	77	5.2 ± 0.9	136	6.1 ± 1.1	138	6.6 ± 1.1	<0.001	<0.001	0.287
Six-minute walk distance (m)	66	699 ± 114	77	629 ± 100	138	554 ± 95	137	525 ± 65	<0.001	<0.001	0.032 *
Normalised jump power (W/kg)	51	48.1 ± 8.2	57	35.6 ± 7.2	97	27.1 ± 5.4	101	21.7 ± 5.1	<0.001	<0.001	<0.001 *
COP-ML_d_	66	0.6 ± 0.1	77	0.5 ± 0.1	89	0.8 ± 0.2	77	0.7 ± 0.2	<0.001	<0.001	0.980
HR%	66	76.7 ± 16.9	77	74.8 ± 15.7	138	80.4 ± 16.2	138	84.1 ± 15.2	<0.001	0.262	0.089
FEV_1_	49	4.7 ± 0.5	55	3.5 ± 0.4	108	2.8 ± 0.5	106	2.1 ± 0.4	<0.001	<0.001	<0.001 *

BMI = body mass index; ALM, appendicular lean muscle mass; COP-ML_d_, root mean square mediolateral sway standing on one leg; HR%, percentage of maximum heart rate attained at the end of the six-minute walk test; FEV_1_, forced expiratory volume in 1 s. * Indicates a significant interaction term. Data are means ± SD.

**Table 2:** Bivariate associations between independent variables, the timed up-and-go test and the six-minute walk distance.

		Young Men			Young Women			Older Men			Older Women		
	Independent variable	*n*	r	*p* value	*n*	r	*p* value	*n*	r	*p* value	*n*	r	*p* value
TUG	Normalised jump power (W/kg)	50	−0.32	0.025	57	0.02	0.894	96	−0.45	<0.001	101	−0.26	0.008
	Height (m)	65	0.19	0.137	77	−0.20	0.084	136	0.04	0.607	138	0.10	0.242
	COP-ML_d_	65	−0.07	0.560	77	−0.11	0.363	89	0.13	0.232	77	−0.01	0.943
	Age (years)	65	0.18	0.158	77	0.14	0.221	136	0.31	<0.001	138	0.26	0.002
	Body mass (kg)	65	0.09	0.459	77	0.05	0.649	136	0.15	0.080	138	0.20	0.016
	BMI (kg·m^−2^)	65	−0.02	0.853	77	0.15	0.179	136	0.14	0.094	138	0.27	0.001
	Fat mass (kg)	64	−0.06	0.648	76	0.16	0.155	132	0.34	<0.001	136	0.31	<0.001
	Lean mass (kg)	64	0.16	0.207	76	−0.07	0.563	132	−0.09	0.315	136	−0.13	0.128
	ALM (kg)	64	0.01	0.934	76	−0.09	0.436	132	−0.11	0.207	136	−0.16	0.058
	ALM/height^2^ (kg/m^2^)	64	−0.11	0.373	76	0.02	0.859	132	−0.18	0.043	136	−0.13	0.142
	Fat mass (%)	64	−0.10	0.413	76	0.20	0.091	132	0.37	<0.001	136	0.35	<0.001
	Lean mass (%)	64	0.13	0.322	76	−0.16	0.171	132	−0.34	<0.001	136	−0.34	<0.001
	ALM%	64	−0.06	0.665	76	−0.18	0.119	132	−0.36	<0.001	136	−0.37	<0.001
	FEV_1_	49	−0.14	0.351	55	−0.11	0.410	108	−0.24	0.014	106	−0.18	0.072
	Leg lean mass (kg)	64	−0.02	0.906	76	−0.06	0.634	132	−0.10	0.264	136	−0.17	0.052
													
	Independent variable	*n*	r	*p* value	*n*	r	*p* value		*r*	*p* value	*n*	r	*p* value
6MWD	Normalised jump power (W/kg)	51	0.18	0.201	57	0.08	0.535	97	0.33	<0.001	101	0.37	<0.001
	Height (m)	66	0.09	0.476	77	0.22	0.059	138	0.03	0.687	137	0.23	0.006
	COP-ML_d_	66	−0.20	0.104	77	0.20	0.081	89	0.00	0.976	76	0.01	0.932
	Age (years)	66	0.18	0.142	77	–0.11	0.351	138	–0.11	0.192	137	–0.27	0.001
	Body mass (kg)	66	−0.08	0.500	77	0.13	0.245	138	–0.17	0.040	137	–0.03	0.733
	BMI (kg·m^−2^)	66	−0.15	0.234	77	0.03	0.818	138	–0.22	0.009	137	–0.17	0.043
	Fat mass (kg)	65	−0.19	0.128	76	0.03	0.805	133	–0.24	0.006	135	–0.13	0.142
	Lean mass (kg)	65	0.04	0.745	76	0.18	0.121	133	0.02	0.814	135	0.32	<0.001
	ALM (kg)	65	0.04	0.748	76	0.13	0.269	133	0.07	0.400	135	0.33	<0.001
	ALM/height^2^ (kg/m^2^)	65	−0.01	0.947	76	0.00	0.994	133	0.06	0.522	135	0.24	0.005
	Fat mass (%)	65	−0.22	0.080	76	–0.03	0.765	133	–0.22	0.009	135	–0.19	0.024
	Lean mass (%)	65	0.23	0.070	76	0.02	0.847	133	0.26	0.002	135	0.27	0.002
	ALM%	65	0.18	0.141	76	–0.01	0.932	133	0.32	<0.001	135	0.33	<0.001
	FEV_1_	49	0.21	0.143	55	0.22	0.113	108	0.31	<0.001	105	0.26	0.007
	Leg lean mass (kg)	65	−0.01	0.908	76	0.10	0.381	133	0.09	0.278	135	0.34	<0.001
	HR%	66	0.42	<0.001	77	0.59	<0.001	138	0.64	<0.001	137	0.65	<0.001

TUG, timed up-and-go; 6MWD, six-minute walk distance; COP-ML_d_, root mean square mediolateral sway standing on one leg; ALM, appendicular lean muscle mass; FEV_1_, forced expiratory volume in 1 s; HR%, percentage of maximum heart rate attained at the end of the six-minute walk test. r = Pearson correlation coefficient.

**Table 3:** Parsimonious multivariate regression models with the timed up-and-go test as the dependent variable and body composition and physiological variables as independent variables.

		Young Men					Young Women					Older Men					Older Women		
*n*	TUG	β	R^2^_adj_	*p*	*n*		β	R^2^_adj_	*p*	*n*		β	R^2^_adj_	*p*	*n*		β	R^2^_adj_	*p*
50	Model 1				77	Model 1				96	Model 1				136	Model 1			
	Normalised jump power (W/kg)	−0.24	0.08	0.025		Height (m)	−0.58	0.03	0.084		Normalised jump power (W/kg)	−0.88	0.19	<0.001		ALM%	−0.92	0.13	<0.001
					76	Model 2				93	Model 2				136	Model 2			
						Height (m)	−0.53	0.04	0.121		Normalised jump power (W/kg)	−0.90	0.29	<0.001		ALM%	−0.82	0.16	<0.001
						Fat mass (%)	0.04		0.125		Fat mass (%)	0.14		<0.001		Age	0.01		0.01
										93	Model 3								
											Normalised jump power (W/kg)	−0.78	0.30	<0.001					
											Fat mass (%)	0.15		<0.001					
											Age	0.01		0.106					

TUG, timed up-and-go; β, standardised coefficient; R^2^_adj_, adjusted R squared; *p*, *p*-value; ALM, appendicular lean muscle mass.

**Table 4:** Explanatory variables - multivariate regression models stratified by age and sex, with the timed up-and-go test as the dependent variable and body composition and physiological variables as independent variables.

		Young Men					Young Women					Older Men					Older Women		
*n*	TUG	β	R^2^_adj_	*p*	*n*		β	R^2^_adj_	*p*	*n*		β	R^2^_adj_	*p*	*n*		β	R^2^_adj_	*p*
50	Model 1				56	Model 1				93	Model 1				100	Model 1			
	Normalised jump power (W/kg)	−0.22	0.07	0.068		Normalised jump power (W/kg)	−0.06	0.06	0.572		Normalised jump power (W/kg)	−0.75	0.30	<0.001		Normalised jump power (W/kg)	−0.30	0.11	0.094
	Height (m)	0.57		0.226		Height (m)	−1.00		0.028		Height (m)	0.49		0.388		Height (m)	0.31		0.589
	Fat mass (%)	−0.03		0.512		Fat mass (%)	0.02		0.520		Fat mass (%)	0.15		<0.001		Fat mass (%)	0.09		0.014
	Age	−0.00		0.876		Age	0.00		0.061		Age	0.01		0.081		Age	0.01		0.145

TUG, timed up-and-go; β, standardised coefficient; R^2^_adj_, adjusted R squared; *p*, *p*-value.

**Table 5:** Parsimonious multivariate regression models with the six-minute walk distance as the dependent variable and body composition and physiological variables as independent variables.

		Young Men					Young Women					Older Men					Older Women		
*n*	6MWD	β	R^2^_adj_	*p*	*n*		β	R^2^_adj_	*p*	*n*		β	R^2^_adj_	*p*	*n*		β	R^2^_adj_	*p*
66	Model 1				77	Model 1				138	Model 1				137	Model 1			
	HR%	0.27	0.17	<0.001		HR%	0.36	0.34	<0.001		HR%	0.40	0.39	<0.001		HR%	0.35	0.39	<0.001
65	Model 2				77	Model 2				97	Model 2				135	Model 2			
	HR%	0.31	0.27	<0.001		HR%	0.35	0.35	<0.001		HR%	0.38	0.43	<0.001		HR%	0.35	0.49	<0.001
	Lean mass (%)	0.34		0.010		COP-ML_d_	0.07		0.010		Normalised jump power (W/kg)	0.24		0.003		ALM%	0.45		<0.001
										94	Model 3				104	Model 3			
											HR%	0.40	0.48	<0.001		HR%	0.32	0.55	<0.001
											Normalised jump power (W/kg)	0.26		0.001		ALM%	0.55		<0.001
											Lean Mass (%)	0.16		0.112		FEV_1_	0.22		0.032
										65	Model 4				104	Model 4			
											HR%	0.29	0.53	<0.001		HR%	0.32	0.60	<0.001
											Normalised jump power (W/kg)	0.34		<0.001		ALM%	0.49		<0.001
											Lean Mass (%)	0.16		0.169		FEV_1_	0.10		0.076
											FEV_1_	0.11		0.102		Age	−0.01		0.001

6MWD, six-minute walk distance; β, standardised coefficient; R^2^_adj_, adjusted R squared; *P*, *P*-value; HR%, percentage of maximum heart rate attained at the end of the six-minute walk test; COP-ML_d_, root mean square mediolateral sway standing on one leg; FEV_1_, forced expiratory volume in 1 s; ALM, appendicular lean muscle mass.

**Table 6:** Explanatory variables - multivariate regression models stratified by age and sex, with the six-minute walk distance as the dependent variable and body composition and physiological variables as independent variables.

		Young Men					Young Women					Older Men					Older Women		
*n*	6MWD	β	R^2^_adj_	*p*	*n*		β	R^2^_adj_	*p*	*n*		β	R^2^_adj_	*p*	*n*		β	R^2^_adj_	*p*
35	Model 1				35	Model 1				42	Model 1				41	Model 1			
	HR%	0.33	0.28	0.004		HR%	0.55	0.39	<0.001		HR%	0.42	0.70	<0.001		HR%	0.26	0.43	<0.001
	Normalised jump power (W/kg)	0.04		0.697		Normalised jump power (W/kg)	−0.19		0.156		Normalised jump power (W/kg)	−0.02		0.883		Normalised jump power (W/kg)	0.23		0.047
	Lean mass (%)	0.22		0.183		Lean mass (%)	0.54		0.008		Lean mass (%)	0.27		0.025		Lean mass (%)	0.15		0.091
	COP-ML_d_	−0.05		0.462		COP-ML_d_	0.05		0.486		COP-ML_d_	−0.03		0.364		COP-ML_d_	0.05		0.058
	FEV_1_	0.14		0.191		FEV_1_	0.06		0.589		FEV_1_	0.07		0.373		FEV_1_	0.08		0.453
	Age	0.00		0.495		Age	0.00		0.747		Age	−0.01		0.001		Age	−0.01		0.027

6MWD, six-minute walk distance; R^2^_adj_, adjusted R squared; *P*, *P*-value; HR%, percentage of maximum heart rate attained at the end of the six-minute walk test; COP-ML_d_, root mean square mediolateral sway standing on one leg; FEV_1_, forced expiratory volume in 1 s.

## References

[1] Podsiadlo D., Richardson S. (1991). The Timed “Up & Go”: A Test of Basic Functional Mobility for Frail Elderly Persons. J. Am. Geriatr. Soc..

[2] Eagles D., Perry J.J., Sirois M.J., Lang E., Daoust R., Lee J., Griffith L., Wilding L., Neveu X., Emond M. (2017). Timed Up and Go predicts functional decline in older patients presenting to the emergency department following minor trauma. Age Ageing.

[3] Sebastião E., Sandroff B.M., Learmonth Y.C., Motl R.W. (2016). Validity of the Timed Up and Go Test as a Measure of Functional Mobility in Persons with Multiple Sclerosis. Arch. Phys. Med. Rehabil..

[4] Huang W.-N.W., Perera S., Vanswearingen J., Studenski S. (2010). Performance measures predict onset of activity of daily living difficulty in community-dwelling older adults. J. Am. Geriatr. Soc..

[5] Northgraves M.J., Hayes S.C., Marshall P., Madden L.A., Vince R.V. (2016). The test-retest reliability of four functional mobility tests in apparently healthy adults. Isokinet. Exerc. Sci..

[6] Steffen T.M., Hacker T.A., Mollinger L. (2002). Age- and Gender-Related Test Performance in Community-Dwelling Elderly People: Six-Minute Walk Test, Berg Balance Scale, Timed Up & Go Test, and Gait Speeds. Phys. Ther..

[7] Mathias S., Nayak U., Isaacs B. (1986). Balance in elderly patients: The “get-up and go” test. Arch. Phys. Med. Rehabil..

[8] Benavent-Caballer V., Sendín-Magdalena A., Lisón J.F., Rosado-Calatayud P., Amer-Cuenca J.J., Salvador-Coloma P., Segura-Ortí E. (2016). Physical factors underlying the Timed “Up and Go” test in older adults. Geriatr. Nurs. (Minneap).

[9] Chen T., Chou L.-S. (2017). Effects of Muscle Strength and Balance Control on Sit-to-Walk and Turn Durations in the Timed Up and Go Test. Arch. Phys. Med. Rehabil..

[10] Cruz-Jentoft A.J., Baeyens J.P., Bauer J.M., Boirie Y., Cederholm T., Landi F., Martin F.C., Michel J.P., Rolland Y., Schneider S.M. (2010). Sarcopenia: European consensus on definition and diagnosis. Age Ageing.

[11] Kang L., Han P., Wang J., Ma Y., Jia L., Fu L., Yu H., Chen X., Niu K., Guo Q. (2017). Timed up and go test can predict recurrent falls: A longitudinal study of the community-dwelling elderly in China. Clin. Interv. Aging.

[12] Nocera J.R., Stegemöller E.L., Malaty I.A., Okun M.S., Marsiske M., Hass C.J. (2013). Using the Timed Up & Go Test in a Clinical Setting to Predict Falling in Parkinson’s Disease. Arch. Phys. Med. Rehabil..

[13] Shumway-Cook A., Brauer S., Woollacott M. (2000). Predicting the Probability for Falls in Community-Dwelling Older Adults Using the Timed Up & Go Test. Phys. Ther..

[14] Khazzani H., Allali F., Bennani L., Ichchou L., Mansouri L.E., Abourazzak F.E., Abouqal R., Hajjaj-Hassouni N. (2009). The relationship between physical performance measures, bone mineral density, falls, and the risk of peripheral fracture: A cross-sectional analysis. BMC Public Health.

[15] Jehu D.A., Paquet N., Lajoie Y. (2017). Balance and mobility training with or without concurrent cognitive training improves the timed up and go (TUG), TUG cognitive, and TUG manual in healthy older adults: An exploratory study. Aging Clin. Exp. Res..

[16] Nightingale C.J., Mitchell S.N., Butterfield S.A. (2018). Validation of the Timed Up and Go Test for Assessing Balance Variables in Adults Aged 65 and Older. J. Aging Phys. Act..

[17] Maden-Wilkinson T.M., McPhee J.S., Jones D.A., Degens H. (2015). Age-related loss of muscle mass, strength, and power and their association with mobility in recreationally-active older adults in the United Kingdom. J. Aging Phys. Act..

[18] Bean J.F., Kiely D.K., Herman S., Leveille S.G., Mizer K., Frontera W.R., Fielding R.A. (2002). The relationship between leg power and physical performance in mobility-limited older people. J. Am. Geriatr. Soc..

[19] Orange S.T., Marshall P., Madden L.A., Vince R.V. (2019). Can sit-to-stand muscle power explain the ability to perform functional tasks in adults with severe obesity?. J. Sports Sci..

[20] Zarzeczny R., Nawrat-Szołtysik A., Polak A., Maliszewski J., Kiełtyka A., Matyja B., Dudek M., Zborowska J., Wajdman A. (2017). Aging effect on the instrumented Timed-Up-and-Go test variables in nursing home women aged 80–93 years. Biogerontology.

[21] Sperandio E.F., Arantes R.L., Matheus A.C., Silva R.P., Lauria V.T., Romiti M., Gagliardi A.R.T., Dourado V.Z. (2015). Intensity and physiological responses to the 6-minute walk test in middle-aged and older adults: A comparison with cardiopulmonary exercise testing. Braz. J. Med. Biol. Res..

[22] Mänttäri A., Suni J., Sievänen H., Husu P., Vähä-Ypyä H., Valkeinen H., Tokola K., Vasankari T. (2018). Six-minute walk test: A tool for predicting maximal aerobic power (VO2max) in healthy adults. Clin. Physiol. Funct. Imaging.

[23] Pasma J.H., Stijntjes M., Ou S.S., Blauw G.J., Meskers C.G.M., Maier A.B. (2014). Walking speed in elderly outpatients depends on the assessment method. Age.

[24] Morley J.E., Abbatecola A.M., Argiles J.M., Baracos V., Bauer J., Bhasin S., Cederholm T., Coats A.J.S., Cummings S.R., Evans W.J. (2011). Sarcopenia With Limited Mobility: An International Consensus. J. Am. Med. Dir. Assoc..

[25] Young S.D., Montes J., Kramer S.S., Marra J., Salazar R., Cruz R., Chiriboga C.A., Garber C.E., De Vivo D.C. (2016). Six-minute walk test is reliable and valid in spinal muscular atrophy. Muscle Nerve.

[26] Cahalin L., Pappagianopoulos P., Prevost S., Wain J., Ginns L. (1995). The relationship of the 6-min walk test to maximal oxygen consumption in transplant candidates with end-stage lung disease. Chest.

[27] Troosters T., Gosselink R., Decramer M. (2002). Six-Minute Walk Test: A Valuable Test, When Properly Standardized. Phys. Ther..

[28] Yazdanyar D.A., Aziz M.M., Enright P.L., Edmundowicz D., Boudreau R., Sutton-Tyrell K., Kuller L., Newman A.B. (2014). Association between Six Minute Walk Test and All-Cause Mortality, Coronary Heart Disease-Specific Mortality, and Incident Coronary Heart Disease. J. Aging Health.

[29] Zou H., Zhang J., Chen X., Wang Y., Lin W., Lin J., Chen H., Pan J. (2017). Reference Equations for the Six-Minute Walk Distance in the Healthy Chinese Han Population, Aged 18–30 Years. BMC Pulm. Med..

[30] Zou H., Zhu X., Zhang J., Wang Y., Wu X., Liu F., Xie X., Chen X. (2017). Reference equations for the six-minute walk distance in the healthy Chinese population aged 18–59 years. PLoS ONE.

[31] Bean J.F., Kiely D.K., Leveille S.G., Herman S., Huynh C., Fielding R., Frontera W. (2002). The 6-minute walk test in mobility-limited elders: What is being measured?. J. Gerontol.-Ser. A Biol. Sci. Med. Sci..

[32] Marsh A.P., Miller M.E., Saikin A.M., Rejeski W.J., Hu N., Lauretani F., Bandinelli S., Guralnik J.M., Ferrucci L. (2006). Lower Extremity Strength and Power Are Associated With 400- Meter Walk Time in Older Adults: The InCHIANTI Study. J. Gerontol. A Biol. Sci. Med. Sci..

[33] Bautmans I., Lambert M., Mets T. (2004). The six-minute walk test in community dwelling elderly: Influence of health status. BMC Geriatr..

[34] Agrawal M.B., Awad N.T. (2015). Correlation between six minute walk test and spirometry in chronic pulmonary disease. J. Clin. Diagn. Res..

[35] Sillanpää E., Stenroth L., Bijlsma A.Y., Rantanen T., McPhee J.S., Maden-Wilkinson T.M., Jones D.A., Narici M.V., Gapeyeva H., Pääsuke M. (2014). Associations between muscle strength, spirometric pulmonary function and mobility in healthy older adults. Age.

[36] Camarri B., Eastwood P.R., Cecins N.M., Thompson P.J., Jenkins S. (2006). Six minute walk distance in healthy subjects aged 55–75 years. Respir. Med..

[37] Xie Y.J., Liu E.Y., Anson E.R., Agrawal Y. (2017). Age-related imbalance is associated with slower walking speed: Analysis from the National Health and Nutrition Examination Survey. J. Geriatr. Phys. Ther..

[38] McPhee J.S., Hogrel J.Y., Maier A.B., Seppet E., Seynnes O.R., Sipilä S., Bottinelli R., Barnouin Y., Bijlsma A.Y., Gapeyeva H. (2013). Physiological and functional evaluation of healthy young and older men and women: Design of the European MyoAge study. Biogerontology.

[39] Onambele G.L., Narici M.V., Maganaris C.N. (2006). Calf muscle-tendon properties and postural balance in old age. J. Appl. Physiol..

[40] Enright P.L. (2003). The Six-Minute Walk Test Introduction Standards and Indications 6-Minute Walk Test Versus Shuttle Walk Test Safety Variables Measured Conducting the Test Ensuring Quality Factors That Influence 6-Minute Walk Distance Interpreting the Results Improving the. Respir. Care.

[41] Fox S.M., Naughton J.P. (1972). Physical Activity and the Prevention of Coronary Heart Disease. Prev. Med. (Baltim).

[42] Caserotti P., Aagaard P., Simonsen E.B., Puggaard L. (2001). Contraction-specific differences in maximal muscle power during stretch-shortening cycle movements in elderly males and females. Eur. J. Appl. Physiol..

[43] Degens H., Maden-Wilkinson T.M., Ireland A., Korhonen M.T., Suominen H., Heinonen A., Radak Z., McPhee J.S., Rittweger J. (2013). Relationship between ventilatory function and age in master athletes and a sedentary reference population. Age (Omaha).

[44] Cormie P., McGuigan M.R., Newton R.U. (2011). Developing maximal neuromuscular power: Part 1 - biological basis of maximal power production. Sports Med..

[45] Reid K.F., Fielding R.A. (2012). Skeletal Muscle Power: A Critical Determinant of Physical. Exerc. Sport Sci. Rev..

[46] Pojednic R.M., Clark D.J., Patten C. (2013). The specific ontributions fo force & velocity to muscle power in Older Adults. Exp. Gerontol..

[47] Arampatzis A., Degens H., Baltzopoulos V., Rittweger J. (2011). Why do older sprinters reach the finish line later?. Exerc. Sport Sci. Rev..

[48] Buchner D.M., Cress M.E., Esselman P.C., Margherita A.J., de Lateur B.J., Campbell A.J., Wagner E.H. (1996). Factors Associated With Changes in Gait Speed in Older Adults. J. Gerontol. Ser. A Biol. Sci. Med. Sci..

[49] Bendall M.J., Bassey E.J., Pearson M.B. (1989). Factors affecting walking speed of elderly people. Age Ageing.

[50] Samson M.M., Meeuwsen I.B.A.E., Crowe A., Dessens J.A.G., Duursma S.A., Verhaar H.J.J. (2000). Relationships between physical performance measures, age, height and body weight in healthy adults. Age Ageing.

[51] Inoue W., Ikezoe T., Tsuboyama T., Sato I., Malinowska K.B., Kawaguchi T., Tabara Y., Nakayama T., Matsuda F., Ichihashi N. (2017). Are there different factors affecting walking speed and gait cycle variability between men and women in community-dwelling older adults?. Aging Clin. Exp. Res..

[52] Bijlsma A.Y., Meskers C.G.M., Van Den Eshof N., Westendorp R.G., Sipilä S., Stenroth L., Sillanpää E., McPhee J.S., Jones D.A., Narici M.V. (2014). Diagnostic criteria for sarcopenia and physical performance. Age (Omaha).

[53] Henwood T.R., Riek S., Taaffe D.R. (2008). Strength versus muscle power-specific resistance training in community-dwelling older adults. J. Gerontol. Med. Sci..

[54] Uematsu A., Hortobágyi T., Tsuchiya K., Kadono N., Kobayashi H., Ogawa T., Suzuki S. (2018). Lower extremity power training improves healthy old adults’ gait biomechanics. Gait Posture.

[55] McClaran S.R., Babcock M.A., Pegelow D.F., Reddan W.G., Dempsey J.A. (1995). Longitudinal effects of aging on lung function at rest and exercise in healthy active fit elderly adults. J. Appl. Physiol..

